# Inclisiran in Dyslipidemia with High Residual Platelet Reactivity

**DOI:** 10.3390/diseases14010030

**Published:** 2026-01-12

**Authors:** Dina Kapsultanova, Sholpan Zhangelova, Friba Nurmukhammad, Zulfiya Makasheva, Orazbek Sakhov, Tamara Galkina, Farida Rustamova, Dana Akhmentayeva, Botakoz Aubakirova

**Affiliations:** 1 Faculty of Postgraduate Education, Asfendiyarov Kazakh National Medical University, Almaty 050012, Kazakhstan; kapsultanova.d@kaznmu.kz (D.K.); zhangelova.s@kaznmu.kz (S.Z.); sakhov.o@kaznmu.kz (O.S.); rustamova.f@kaznmu.kz (F.R.); 2 Faculty of Postgraduate Medical Education, Hodja Ahmed Yasawi International Kazakh-Turkish University, Turkestan 161200, Kazakhstan; 3 Department of Cardiology, City Cardiology Center, Almaty 050000, Kazakhstanaubakirova.botakoz@mail.ru (B.A.); 4 Department of Cardiology, Medical Center «AMAST», Astana 010000, Kazakhstan; galchenoktamara@mail.ru

**Keywords:** high residual platelet reactivity, revascularization, LDL cholesterol, inclisiran, platelet function testing, familial hypercholesterolemia

## Abstract

Background: High residual platelet reactivity (HRPR) and persistent dyslipidemia remain important unmet needs in cardiovascular risk management, particularly in patients undergoing coronary revascularization. Despite intensive lipid-lowering and antiplatelet therapy, a substantial proportion of patients fail to reach recommended low-density lipoprotein cholesterol (LDL-C) targets or exhibit inadequate platelet inhibition. Inclisiran, a PCSK9-targeting small interfering RNA, represents an emerging approach for long-term LDL-C reduction. Methods: A narrative review of the literature published between 2009 and 2025 was performed using PubMed, Scopus, Web of Science, and MEDLINE. Studies evaluating the addition of inclisiran to standard lipid-lowering therapy in patients with dyslipidemia and HRPR, assessed using the VerifyNow assay, were included. Illustrative clinical cases from Kazakhstan were analyzed to demonstrate real-world changes in LDL-C levels and platelet reactivity following insufficient response to conventional treatment. The review had a descriptive design. Results: Available evidence indicates that a significant proportion of high- and very-high-risk patients do not achieve LDL-C targets or are unable to tolerate high-intensity statin therapy. Inclisiran consistently induces sustained reductions in LDL-C and circulating PCSK9 levels. Emerging data suggest a potential indirect modulation of platelet reactivity associated with intensive lipid lowering. In patients at extreme cardiovascular risk—including those after coronary artery bypass grafting (CABG) and with long-standing multivessel coronary artery disease—inclisiran therapy was associated with marked LDL-C reduction and a trend toward normalization of platelet reactivity. Conclusions: Assessment of platelet function using the VerifyNow assay may improve identification of residual thrombotic risk in patients with advanced atherosclerotic disease. Inclisiran appears to be a promising adjunctive therapy for dyslipidemic patients with persistently elevated cardiovascular risk and HRPR despite standard treatment. Further prospective studies are warranted to clarify the relationship between intensive LDL-C lowering, platelet reactivity, and clinical outcomes, and to optimize integrated lipid-lowering and antiplatelet strategies.

## Simple Summary

Despite modern medical treatment, many patients with cardiovascular disease remain at high risk of heart attacks and strokes. This risk is often linked to two persistent problems: high levels of harmful cholesterol and excessive activity of blood platelets, which can promote clot formation even during standard therapy. This review aimed to summarize available evidence and real-world clinical examples evaluating a novel cholesterol-lowering treatment added to conventional care in such high-risk patients. Published studies and patient observations show that this therapy can lead to substantial and sustained reductions in harmful cholesterol levels in individuals who do not reach treatment goals with commonly used drugs. In addition, emerging data suggest that improved cholesterol control may be accompanied by normalization of platelet activity in selected patients with very high cardiovascular risk. Clinical examples illustrate that patients with advanced coronary disease achieved better control of both cholesterol levels and platelet behavior after treatment intensification. These findings highlight the importance of individualized risk assessment using platelet function testing and modern lipid-lowering approaches. Improving control of these key risk factors may help prevent future cardiovascular events and reduce the long-term burden of cardiovascular disease on patients and healthcare systems.

## 1. Introduction

According to a large population-based study conducted in Kazakhstan in 2022, 43.5% of the population aged 18–69 years had dyslipidemia. Moreover, the prevalence increased with age, reaching approximately 71% in the 60–69-year age group [1]. According to World Health Organization data reported until recent years, approximately 12% of the adult population in Kazakhstan had elevated total cholesterol levels (≥6.2 mmol/L). In a cross-sectional study of adults aged 50–74 years in Astana and surrounding areas, hypercholesterolemia (total cholesterol ≥ 6.2 mmol/L) was present in ~37%, with fewer than half aware of their condition and only some receiving treatment [2]. Among post-coronary artery bypass grafting patients in Kazakhstan, high on-treatment platelet reactivity was observed in ~45% overall and ~66% among those undergoing repeat coronary intervention [3]. Across different studies, the prevalence of HTPR (high on-treatment platelet reactivity) ranged from approximately ~6% to 60% of patients, depending on the platelet function tests used and the studied populations [4]. A review of patients with peripheral arterial disease demonstrated that the prevalence of HTPR varied from ~9.8% to 77% in patients receiving clopidogrel and from ~4.1% to 50% in those treated with acetylsalicylic acid [5].

Atherosclerosis development is strongly influenced by dyslipidemia, a modifiable cardiovascular risk factor. In addition to lifestyle interventions, lipid-lowering therapy remains for acute coronary syndromes, which are among the leading causes of death. While cholesterol reduction improves outcomes, 10–20% of high- and very-high-risk patients fail to achieve fundamental reductions in cardiovascular risk [6].

Chronic hypercholesterolemia contributes to the initiation and progression of atherosclerosis, affecting coronary, carotid and peripheral arteries, and directly damaging the myocardium. Globally, atherosclerosis and its complications, including recommended LDL-C targets with statins, highlight the need for additional therapeutic strategies [7,8]. Lipid accumulation within the vascular wall is central to atherogenesis, and elevated plasma LDL-C levels are a well-established cardiovascular risk factor. For very-high-risk patients, including those with familial hypercholesterolemia who do not achieve LDL-C targets on maximal statin and ezetimibe therapy, the addition of a PCSK9 inhibitor is recommended [7,9,10]. Familial hypercholesterolemia (FH) is the most common inherited disorder of lipid metabolism. It follows an autosomal dominant pattern and is characterized by elevated LDL-C, leading to a markedly increased risk of premature cardiovascular disease. Clinical manifestations include xanthomas (commonly on the elbows, Achilles tendon, and hands), xanthelasmas around the eyes and presenile corneal arcus. Regular screening for subclinical aortic, carotid and coronary disease is therefore advised. PCSK9 inhibitors reduce LDL-C and lipoprotein(a) [Lp(a)] levels by 25–30%, which may further reduce cardiovascular risk [11,12]. Heterozygous FH accounts for up to 10% of premature acute coronary syndromes. CABG is a widely used revascularization procedure for patients with severe coronary artery disease (CAD) and is associated with low morbidity and mortality [13]. However, revascularization/CABG triggers significant activation of the hemostatic system, with platelets playing a central role in thrombotic complications. Postoperative alterations in platelet indices, including platelet count and mean platelet volume, indicate enhanced platelet activity [13,14]. Approximately 21% of patients undergoing Percutaneous Coronary Intervention later require CABG, and nearly 13% of CABG patients are on clopidogrel or other ADP inhibitors at the time of surgery [15].

HRPR predicts adverse cardiovascular events and may accelerate atherogenesis, particularly when accompanied by elevated LDL-C [16,17,18]. Inclisiran is a small interfering RNA (siRNA) therapy targeting PCSK9. Conjugated to N-acetylgalactosamine, it selectively enters hepatocytes and degrades PCSK9 mRNA, reducing PCSK9 production and enhancing LDL receptor expression. The PCSK9-REACT study demonstrated that elevated PCSK9 levels in acute coronary syndrome patients on dual antiplatelet therapy are associated with increased platelet reactivity, reduced antiplatelet efficacy and higher risk of major cardiovascular events [19]. High-intensity statins remain first-line therapy, yet approximately 80% of patients with atherosclerotic cardiovascular disease do not achieve LDL-C targets [20]. In clinical trials, two doses of 284 mg inclisiran (equivalent to 300 mg inclisiran sodium) administered on day 1 and day 90 reduced LDL-C by 52.6% at 180 days [21]. Inclisiran was approved in the European Union in 2020 for adults with primary hypercholesterolemia or mixed dyslipidemia [22]. Here, we present two clinical cases of post-CABG patients with comorbid dyslipidemia and HRPR who were unresponsive to monotherapy and dual therapy with statins and ezetimibe. Both patients received inclisiran, resulting in effective LDL-C reduction and normalization of platelet reactivity. One patient exhibited corneal arcus and xanthelasmas, with markedly elevated LDL-C, suggesting possible familial hypercholesterolemia. Platelet normalization may be attributed to reduced LDL-C and PCSK9 levels, which are implicated in HRPR development. These cases highlight the clinical relevance of monitoring platelet activity and the potential role of inclisiran in high-risk patients with coexisting dyslipidemia and HRPR, aiming to improve long-term cardiovascular outcomes.

## 2. Patient-Level Examples of High Residual Platelet Reactivity During Inclisiran Therapy

### 2.1. Patient 1

A 49-year-old male was referred to the Scientific Research Institute of Cardiology and Internal Diseases, Almaty, on 22 January 2024, presenting with pressing retrosternal chest pain radiating to the interscapular region lasting 15–20 min, shortness of breath on minimal exertion, general weakness, headache, and dizziness.

Medical History: The patient had type 2 diabetes mellitus, treated with metformin, canagliflozin 300 mg, and liraglutide 6 IU, but glycemic control remained suboptimal. Cardiovascular history included ischemic heart disease, multiple myocardial infarctions (2012, 2016, 2017, 2018, 2019), percutaneous coronary interventions with stent placements in the right coronary artery (2012, 2016) and circumflex branch (2019), and CABG in 2017. There was no history of smoking, alcohol use, or familial cardiovascular disease.

Family History: Father with a history of myocardial infarction; no other significant cardiovascular diseases reported among relatives. No evidence of FH or prior cardiac surgery in the family.

Physical Examination and Laboratory Findings: Physical examination revealed no xanthelasmas, xanthomas, or corneal arcus; mild lower-extremity edema was noted. Laboratory results indicated dyslipidemia: total cholesterol 7.83 mmol/L, LDL-C 5.31 mmol/L, triglycerides 5.44 mmol/L.

Medication at Presentation: The patient was on clopidogrel 75 mg, spironolactone 50 mg, ramipril 10 mg, bisoprolol 2.5 mg, omeprazole 20 mg, acetylsalicylic acid 100 mg, and rosuvastatin 20 mg.

Platelet Reactivity: Residual platelet reactivity (RPR) was assessed using the VerifyNow P2Y12 assay (Instrumentation Laboratory, Bedford, MA, USA), which measures platelet inhibition and reports results as P2Y12 Reaction Units (PRU). HRPR was observed with a PRU of 245 in January 2024. PRU values of 95–208 are considered normal. HRPR was defined as PRU ≥ 208.

Given the patient’s extreme cardiovascular risk profile—including five previous myocardial infarctions, CABG, type 2 diabetes, HRPR, and elevated LDL-C (up to 5.31 mmol/L)—therapy with inclisiran was recommended.

Blood Collection Procedure for Assessment of Platelet Reactivity: Blood sampling was performed 2 h after the administration of antiplatelet therapy. Blood collection tubes for the VerifyNow (P2Y12) assay contain 3.2% sodium citrate, which is used to prevent coagulation and to ensure accurate assessment of platelet aggregation. The collection tube was filled to the designated mark and then inverted five times to ensure proper distribution of blood within the cartridge. Subsequently, the tube was placed into the VerifyNow analyzer. The result was displayed on the analyzer screen within 3–5 min.

Treatment and Follow-Up: The patient continued rosuvastatin 20 mg and received the first inclisiran injection on 26 March 2024. Lipid profile and biochemical parameters were monitored every 3–6 months (Table 1). Inclisiran was provided free of charge to a patient as part of a study conducted by the Committee of Science of the Ministry of Science and Higher Education of the Republic of Kazakhstan “Improving the outcomes of surgical myocardial revascularization based on the development of comprehensive innovative management of patients after intervention”, grant number “IRN AR19680319”.

Following rosuvastatin therapy alone, LDL-C decreased to 2.2 mmol/L by March 2024, but the target of <1.0 mmol/L for extreme-risk patients was not achieved. After inclisiran administration, LDL-C decreased to 1.29 mmol/L by July 2024, achieving the very high-risk target, and further decreased to 0.58 mmol/L by November 2024, meeting the extreme-risk target. Residual platelet reactivity also normalized (PRU 190). The patient reported no adverse effects.

Summary: Inclisiran therapy resulted in a significant reduction in LDL-C, achieving guideline-recommended targets for very high- and extreme-risk patients. Improvements were also observed in total cholesterol, apolipoprotein B, and lipoprotein(a), with sustained efficacy over several months. Glycemic control remained suboptimal, and the patient was advised to consult an endocrinologist for diabetes management.

### 2.2. Patient 2

A 54-year-old male was referred to the cardiology department in Astana on 4 November 2025, presenting with nocturnal chest discomfort radiating to the back, relieved by rest and lasting approximately 5 min, and shortness of breath with moderate exertion.

Medical History: The patient was diagnosed with familial dyslipidemia in June 2025 and initiated inclisiran therapy (284 mg) on 5 June 2025, with a second injection on 5 September 2025. Diagnostic coronary angiography on 30 July 2025 revealed single-vessel coronary artery disease. High residual platelet reactivity (HRPR) was detected, with a PRU of 230.

Family History: Family history was significant for early cardiovascular disease: a brother underwent stenting at age 45, and the father died suddenly at age 55. The patient’s brother was also diagnosed with FH and was prescribed optimal lipid-lowering therapy. There was no history of smoking or alcohol use.

Physical Examination and Laboratory Findings: Examination revealed xanthelasmas around the eyes. Laboratory results indicated dyslipidemia: total cholesterol 7.3 mmol/L, LDL-C 5.0 mmol/L, triglycerides 2.6 mmol/L, and markedly elevated lipoprotein(a) [Lp(a)] at 401 mg/dL.

Medication at Presentation: The patient was prescribed nebivolol 2.5 mg, acetylsalicylic acid 100 mg, atorvastatin 20 mg, ramipril 2.5 mg, ezetimibe 10 mg, and pantoprazole 20 mg.

Platelet Reactivity: Residual platelet reactivity (RPR) was assessed using the VerifyNow P2Y12 assay (Instrumentation Laboratory, Bedford, MA, USA). HRPR was present, with a PRU of 178 on 4 November 2025.

Diagnosis: According to the Dutch Lipid Clinic Network (DLCN) criteria, a score of 9 indicated a definite diagnosis of heterozygous FH. Genetic testing was unavailable locally; therefore, mutations in *LDLR*, *APOB*, or *PCSK9* could not be confirmed.

Treatment and Follow-Up: Despite dual therapy with statins and ezetimibe, the patient did not achieve LDL-C targets, prompting initiation of triple therapy with inclisiran. Lipid profile and biochemical parameters were monitored every three months (Table 2).

Following triple therapy with atorvastatin, ezetimibe, and inclisiran, the patient achieved LDL-C targets, with a notable 25% reduction in Lp(a) and normalization of triglycerides. Platelet reactivity also improved, indicating normalization of HRPR. These results demonstrate the efficacy of inclisiran in a high-risk patient with heterozygous familial hypercholesterolemia and dyslipidemia. Improvement in platelet function suggests potential benefits for long-term cardiovascular prognosis.

The primary cause of HRPR in these patients may be polymorphism of the CYP2C19 gene, leading to resistance to antiplatelet therapy. Moreover, a history of diabetes mellitus may contribute to HRPR due to increased expression of P2Y12 receptors, P-selectin and enhanced platelet aggregation. In addition, multiple episodes of myocardial infarction, a history of coronary stenting and CABG may result in chronic vascular inflammation and persistent platelet activation. Elevated lipid levels also promote inflammation and platelet hyperreactivity. Age (49, 54 years) itself is associated with increased platelet reactivity. The prescription of multiple medications may lead to polypharmacy, which can negatively affect adherence to antiplatelet and lipid-lowering therapies. Furthermore, proton pump inhibitors, particularly omeprazole, may reduce the effectiveness of antiplatelet therapy. Based on these clinical cases, it can be concluded that multiple interacting factors contribute to the development of high residual platelet reactivity.

According to the presented clinical cases, normalization of biochemical markers was observed at the early stage of therapy, with the exception of LDL-C. This finding may be related to the insufficient lipid-lowering efficacy of rosuvastatin at a dose of 20 mg in combination with ezetimibe 10 mg. In addition, the introduction of inclisiran into the treatment regimen did not result in an immediate reduction in LDL-C levels, as the maximal therapeutic effect is typically observed 2–3 months after the first injection. Consequently, biochemical parameters sensitive to inflammatory activity or organ function tend to improve earlier than LDL-C levels.

Moreover, PCSK9 reduction induced by inclisiran may indirectly decrease platelet aggregation, which could explain the normalization of HRPR after treatment. However, the additional administration of antiplatelet therapy may also account for the observed normalization of platelet reactivity units. Therefore, it is not possible to definitively attribute the improvement in platelet function to inclisiran alone, and further studies are required to clarify its potential effects on platelet activity.

## 3. Literature Search Strategy

The review was conducted in accordance with the Preferred Reporting Items for Systematic Reviews and Meta-Analyses (PRISMA) guidelines. A structured literature search was conducted in PubMed, Scopus, Web of Science, and Medline to identify studies on HRPR, dyslipidemia and the use of inclisiran after CABG. The search period spanned from 2009 to 2025. Both free-text and Medical Subject Headings (MeSH) terms were used, including “high platelet reactivity,” “inclisiran,” “high platelet reactivity CABG,” and “residual platelet reactivity.” Boolean operators (AND, OR) were applied to refine the search. Reference lists of relevant studies and reviews were manually screened to identify additional eligible reports.

Screening. Titles and abstracts were independently screened by reviewers to assess relevance. Studies clearly unrelated to dyslipidemia, non-statin therapy, or cardiovascular disease were excluded at this stage.

Eligibility. Full-text articles were retrieved and assessed for eligibility based on predefined inclusion and exclusion criteria. Eligible studies included adult populations with dyslipidemia in the context of atherosclerosis-associated disease and/or coronary heart disease. Both observational studies (cross-sectional, cohort, case–control) and interventional studies, including all available randomized controlled trials, were considered. Particular attention was paid to the scientific novelty, methodological rigor, and reliability of reported outcomes, especially in studies assessing LDL-C levels and platelet reactivity. Studies were excluded if they were non-original publications (editorials, reviews, commentaries, letters), focused exclusively on non-dyslipidemic populations, or provided insufficient or incomplete data, particularly in small sample sizes.

Inclusion criteria comprised clinical cases, case series, narrative reviews and meta-analyses reporting inclisiran use in patients with dyslipidemia and HRPR after percutaneous coronary interventions/CABG, or in those with FH, including pharmacodynamic and safety data. Studies reporting achievement of LDL-C, lipoprotein(a) [Lp(a)] and triglyceride targets were also included.

Exclusion criteria included studies on atrial fibrillation, coagulopathies, editorials and duplicate publications.

Data Extraction and Quality Assessment. Data extraction was performed independently by three reviewers using a standardized data collection form. Study quality was assessed using the Newcastle–Ottawa Scale for observational studies and the Cochrane Risk of Bias Tool for interventional trials. Any discrepancies were resolved through discussion and consensus with a third reviewer.

**Figure 1:**
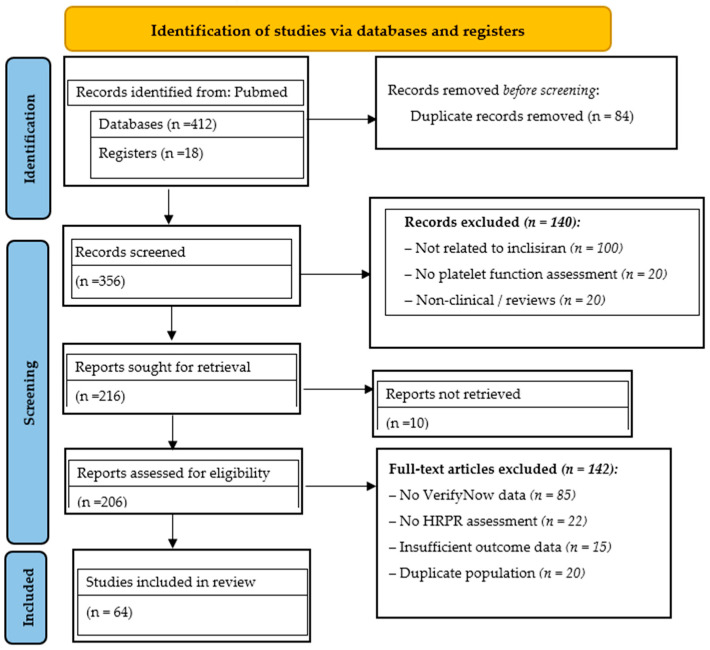
The PRISMA flow diagram.

This strategy ensured the inclusion of well-documented evidence on inclisiran efficacy in HRPR and dyslipidemia post-CABG and in hereditary dyslipidemia. The findings provide comprehensive insights into inclisiran’s effects on LDL-C, Lp(a), triglycerides and platelet reactivity in high-risk post-CABG patients receiving mono- or dual lipid-lowering therapy (statins ± ezetimibe), supporting improved diagnostic assessment and therapeutic decision-making in this comorbid population. The selection process is illustrated in the PRISMA flow diagram (Figure 1).

## 4. Literature Review

LDL-C is a key contributor to atherosclerotic cardiovascular disease, promoting vascular damage and endothelial dysfunction. The Framingham Heart Study demonstrated a 12–13-fold increased risk of acute myocardial infarction in individuals with total cholesterol > 260 mg/dL and HDL-C < 40 mg/dL. Statins remain first-line therapy for lipid disorders, providing both primary and secondary cardiovascular prevention. However, real-world data indicate substantial treatment gaps. The DA VINCI study reported that only 9% of patients received ezetimibe and 1% received PCSK9 inhibitors, despite elevated LDL-C, highlighting the underutilization of combination or advanced lipid-lowering therapies [23]. Globally, approximately 53% of adults have elevated LDL-C, yet less than half receive therapy and only 35% achieve LDL-C control, doubling their risk of atherosclerotic cardiovascular disease (ASCVD). Clinically, PCSK9 levels increase rapidly after acute cardiovascular events, such as acute coronary syndromes, due to its role in regulating LDL receptor degradation in hepatocytes.

Inclisiran, a small interfering RNA targeting PCSK9, reduces its production and can lower LDL-C by over 50% [24]. Ezetimibe, which limits intestinal cholesterol absorption, is more effective when combined with statins. Current guidelines recommend adding ezetimibe to statin therapy when LDL-C goals are not achieved [24,25].

Platelet hyperreactivity is common in the perioperative period. Approximately 75% of patients with platelet hyperreactivity prior to surgery, defined by thrombelastography maximal amplitude (TEG-MA) above reference limits, remain hyperactive one month post-CABG. Both on-pump CABG and off-pump CABG (OPCAB) patients exhibit increased platelet reactivity by postoperative day 4, indicating similar risks of hyperreactivity in both surgical approaches [26]. Adherence to statins is a challenge, with up to 50% of patients discontinuing therapy within one year due to side effects such as myalgia, headache and fatigue. In high-risk patients, inclisiran significantly reduces LDL-C after 180 days compared with placebo [27]. Post-CABG patients may show transient aspirin hyporesponsiveness, though its clinical significance remains uncertain. High on-aspirin residual platelet reactivity (RPR) occurs transiently after CABG and is influenced by factors such as BMI ≥ 27 kg/m^2^, which may necessitate dose adjustments in obese patients [28,29]. Inclisiran is indicated for hypercholesterolemia, dyslipidemia and FH [30]. Its effects differ when used alone versus with statins, as statins upregulate PCSK9 expression. Inclisiran monotherapy reduces LDL-C more effectively than placebo or ezetimibe in patients without ASCVD, diabetes, or FH [31]. Multiple factors influence platelet reactivity and aspirin responsiveness, including age, sex, ethnicity, obesity, hypertension, renal and cardiovascular comorbidities, endothelial dysfunction and inflammation (elevated CRP, IL-6, fibrinogen). Patients with high residual platelet reactivity face a fourfold increased risk of cardiovascular events compared with normal responders [32]. High platelet reactivity is associated with more severe coronary atherosclerosis, with mechanisms including enhanced platelet–endothelial interactions, thrombus formation and accelerated plaque development [33,34]. FH, diagnosed using Dutch Lipid Clinic criteria, requires LDL-C reduction > 50% and below 1.4 mmol/L for high-risk patients. Available therapies include statins, resins, fibrates, niacin, cholesterol absorption inhibitors, PCSK9 inhibitors and siRNA therapies such as inclisiran [35,36]. Inclisiran increases hepatic LDL receptor expression, enhancing LDL-C clearance without significant effects on heart rate or electrocardiographic parameters. Meta-analyses suggest a lower risk of new-onset diabetes compared with atorvastatin, though further studies on glycemic control are warranted [37,38]. Inclisiran reduces myocardial infarction risk but has limited impact on stroke or major adverse cardiovascular events (MACE) in high-risk ASCVD. It is also suitable for statin-intolerant patients, including those with rhabdomyolysis [39,40]. LDL-C lowering reduces cardiovascular risk by approximately 22% per 1 mmol/L reduction. Ezetimibe is recommended for patients intolerant to statins or failing to achieve LDL-C targets. Achieving very low LDL-C levels significantly reduces major cardiovascular events. The IMPROVE-IT trial demonstrated that adding ezetimibe to statins lowered LDL-C by 53.2 mg/dL and improved outcomes. Inclisiran provides sustained reductions in apolipoprotein B, non-HDL-C (non-high-density lipoprotein cholesterol), VLDL-C (very-low-density lipoprotein cholesterol) and TG (triglycerides) over 210 days, with the added advantage of twice-yearly dosing, which may improve adherence while achieving LDL-C reductions comparable to PCSK9 monoclonal antibodies combined with maximal statin or ezetimibe therapy [41,42,43].

## 5. Discussion

Atherosclerosis is a chronic inflammatory arterial disease characterized by lipid imbalance and immune cell infiltration, with acute events such as myocardial infarction and stroke resulting from plaque rupture. Platelets exacerbate endothelial dysfunction and plaque instability, and their interaction with endothelial cells promotes heightened activation and turnover [44]. Dyslipidemia is a modifiable risk factor for both primary and secondary cardiovascular disease (CVD), and its treatment reduces the risk of first or recurrent events. Statins remain first-line therapy, while ezetimibe and PCSK9 monoclonal antibodies provide additional LDL-C reduction and cardiovascular risk mitigation in patients with established ASCVD [45]. Atherosclerosis involves endothelial dysfunction, impaired coronary circulation, vascular smooth muscle cell migration and proliferation, increased matrix metalloproteinase activity, localized inflammation, and alterations in endothelial progenitor cell function. Despite these mechanisms, the relationship between platelet reactivity and cardiovascular risk remains complex and is still under investigation [46]. PCSK9 regulates LDL-C, a major atherosclerosis risk factor, and contributes to plaque progression through lipid accumulation, inflammation, and apoptosis.

Inclisiran, a PCSK9-targeting small interfering RNA (siRNA), provides a novel alternative to monoclonal antibody therapy. Dyslipidemia is typically marked by elevated total cholesterol (TC), triglycerides (TG), and LDL-C, alongside reduced HDL-C [47,48,49,50]. This report presents two rare clinical cases of patients with dyslipidemia and high on-treatment platelet reactivity (HTPR), including one patient with probable familial hypercholesterolemia (FH) and post-revascularization status. Both patients failed to achieve LDL-C targets despite mono- or dual therapy with statins and ezetimibe. Initiation of inclisiran therapy resulted in effective LDL-C reduction and normalization of platelet reactivity. These outcomes may be partially attributed to concurrent antiplatelet therapy but underscore the potential of inclisiran in optimizing lipid control and platelet function.

Platelets from FH individuals are more sensitive than those of non-FH individuals to aggregate in response to adrenaline [51,52]. This finding suggests that FH individuals may develop a greater thrombotic response to stress than non-FH individuals. Extremely high levels of fibrinogen, a primary clotting factor and risk factor for coronary heart disease (CHD) [53], are found in homozygous FH individuals, who have a high incidence of early CHD-related mortality [54]. High levels of fibrinogen also distinguish the subset of heterozygous FH individuals (as well as non-FH individuals) with CHD from those without CHD [55,56]. Genetic factors can influence hemostatic balance. For example, prothrombotic gene polymorphisms, such as prothrombin 20210A, increase the risk of myocardial infarction in the general population [57]. FH individuals with the prothrombin 20210A polymorphism exhibited more than twice the rate of coronary events compared with FH individuals without the polymorphism, an effect that was independent of LDL levels [58]. FH smokers exhibit a shift in hemostatic balance toward thrombosis compared with FH non-smokers. Antoniades et al. demonstrated that FH smokers exhibited a decreased forearm vasodilatory response to reactive hyperemia, increased inflammation, and an imbalanced thrombosis–fibrinolysis equilibrium favoring hypercoagulation compared with FH non-smokers [59]. Sebestjen et al. investigated biomarkers of hypofibrinolysis in FH individuals with and without CHD and found significantly higher levels of tissue plasminogen activator (t-PA) antigen and plasminogen activator inhibitor-1 (PAI-1) antigen—both suppressors of fibrinolysis—in FH individuals with CHD. This shift in hemostatic balance from fibrinolysis toward hypercoagulation was independent of LDL levels [60]. Individuals with FH who die prematurely from CVD appear to be genetically susceptible to developing coagulopathy independent of LDL-C levels [58,61,62,63], as reviewed elsewhere [62,64].

Our findings highlight the importance of assessing residual platelet reactivity using the VerifyNow system in very high-risk patients with dyslipidemia to stratify cardiovascular risk and guide personalized treatment strategies, including the initiation of inclisiran and optimization of antiplatelet therapy. Further research, particularly large-scale case series and systematic reviews, is needed to better define the impact of inclisiran on residual platelet reactivity in patients with multivessel coronary disease and to develop evidence-based lipid-lowering management strategies. Such studies would strengthen the clinical evidence base and support improved care for patients with these uncommon but clinically significant comorbidities.

This report contributes to the limited body of evidence on the coexistence of dyslipidemia and HTPR in post-revascularization patients by documenting the efficacy of inclisiran in these scenarios. By consolidating rare but clinically relevant cases, it raises awareness among interventional cardiologists and lipidologists, promoting improved diagnosis, platelet reactivity assessment, and tailored management approaches. Emerging evidence suggests that elevated LDL-C and PCSK9 levels may contribute to HTPR and that reduction in these factors may enhance platelet responsiveness. In this context, inclisiran therapy in post-CABG patients with dyslipidemia and HTPR may improve both LDL-C levels and platelet reactivity, as demonstrated in our cases, warranting further investigation.

The results from these two clinical cases are limited by the small number of patients with rare and severe forms of dyslipidemia and high platelet reactivity. This restricts the generalizability of the findings to a broader population of patients with more typical dyslipidemia. The effects of inclisiran on LDL-C levels and platelet function may also be influenced by concomitant antiplatelet therapy and individual factors, including genetics and lifestyle.

Patients with FH represent an extremely high cardiovascular risk; therefore, these findings cannot be directly applied to patients with low or moderate risk. These limitations highlight the need for large multicenter studies to evaluate the efficacy of inclisiran in a more diverse patient population. Consequently, the presented data should be considered a stimulus for further research rather than a basis for widespread clinical application.

## 6. Limitations

This report has several limitations. First, the evidence is based on only two clinical cases, which precludes any causal inference and limits generalizability. Second, both cases were managed in routine clinical practice rather than within a standardized research protocol, resulting in potential variability in laboratory measurements, timing of assessments and concomitant therapies that may confound the observed effects. Third, the follow-up period was relatively short, preventing evaluation of long-term lipid-lowering durability, sustained changes in platelet reactivity and rare adverse events. Fourth, no mechanistic data—such as PCSK9 suppression biomarkers, pharmacokinetic parameters, or genetic testing related to antiplatelet response—were collected, leaving the biological links between inclisiran therapy and platelet reactivity speculative. Finally, as both cases originate from Kazakhstan, regional healthcare system characteristics may further limit external applicability.

## 7. Conclusions

Lipid-lowering therapy remains the cornerstone in the management of dyslipidemia; however, a subset of patients fail to achieve target LDL-C levels or develop adverse effects, such as myalgia or elevated liver enzymes, during treatment with statins and ezetimibe. Non-statin therapies, including inclisiran, offer an effective alternative for lowering LDL-C and PCSK9 levels. Emerging evidence indicates that elevated LDL-C and PCSK9 may contribute to HTPR, and their reduction may be associated with improvements in platelet function. In this context, inclisiran therapy in patients with HTPR and dyslipidemia following revascularization/CABG may normalize both LDL-C levels and potentially platelet reactivity, as observed in our cases, highlighting a potential dual benefit that warrants further investigation.

Inclisiran-induced PCSK9 reduction may contribute to HRPR normalization; however, concomitant antiplatelet therapy precludes attributing this effect to inclisiran alone, warranting further study.

Documenting these rare clinical cases expands the current evidence base, increases clinical awareness and facilitates timely identification of patients with HTPR who are unresponsive to standard lipid-lowering therapy. Moreover, assessment of platelet reactivity using the VerifyNow system in post-revascularization patients with dyslipidemia is essential for identifying high-risk individuals and guiding personalized therapeutic strategies. For clinicians, careful optimization of dyslipidemia management and early detection of HTPR are critical for preventing adverse cardiovascular outcomes. Such approaches not only improve patient prognosis but also contribute to a deeper understanding of this uncommon but clinically significant combination of conditions and its therapeutic management.

## Figures and Tables

**Table 1:** Patient 1: Dynamics of the Lipid Profile and Biochemical Parameters After Inclisiran Administration.

Parameter	29 January 2024 Before Rosuvastatin	26 March 2024 First Inclisiran Injection	1 July 2024 Second Inclisiran Injection	7 November 2024 Three Months After Second Injection	Unit
Total cholesterol	7.83	3.70	2.82	2.53	mmol/L
LDL-C	5.31	2.20	1.29	0.58	mmol/L
HDL-C	1.44	0.80	0.85	0.88	mmol/L
Triglycerides	5.44	4.30	3.35	5.60	mmol/L
Apolipoprotein B	-	1.35	1.22	0.53	g/L
Lipoprotein (a)	-	60.0	28.4	16.8	nmol/L
CRP	1.77	5.80	1.09	1.39	mg/L
ALAT	51.6	23.3	26.3	23.9	U/L
ASAT	24.6	16.8	12.6	17.6	U/L
Creatinine	91.49	82.0	92.0	71.0	µmol/L
HbA1c	9.5	10.8	11.2	11.4	%

CRP—C-reactive protein, ALAT—Alanine Aminotransferase, ASAT—Aspartate Aminotransferase, HbA1c—Hemoglobin A1c.

**Table 2:** Patient 2: Dynamics of the Lipid Profile and Biochemical Parameters After Inclisiran Administration.

Parameter	5 June 2025 First Inclisiran Injection	5 September 2025 Second Inclisiran Injection	Unit
Total cholesterol	7.8	3.7	mmol/L
LDL-C	5.0	1.2	mmol/L
HDL-C	1.3	1.0	mmol/L
Triglycerides	2.6	1.4	mmol/L
Lipoprotein (a)	401	300	mg/dL
ALAT	57	25	U/L
ASAT	39.5	24.3	U/L
Creatinine	116	90	µmol/L
HRPR (PRU)	230	178	PRU

LDL-C—Low-density lipoprotein cholesterol, HDL-C—High-Density Lipoprotein Cholesterol, ALAT—Alanine Aminotransferase, ASAT—Aspartate Aminotransferase, HRPR—High Residual Platelet Reactivity, PRU—P2Y12 Reaction Units.

## Data Availability

The authors confirm that the information supporting the findings of this study are available within the article.

## Abbreviations

The following abbreviations are used in this manuscript:

HRPR	High Residual Platelet Reactivity
ASCVD	Atherosclerotic cardiovascular disease
FH	Familial hypercholesterolemia
CABG	Coronary artery bypass grafting
CAD	Coronary artery disease
ALAT	Alanine Aminotransferase
ASAT	Aspartate Aminotransferase
CVD	Cardiovascular disease
OPCAB	Of pump coronary artery bypass grafting
TEG-MA	Thrombelastography maximal amplitude
HDL-C	High-Density Lipoprotein Cholesterol
HTPR	High on-Treatment Platelet Reactivity
LDL-C	Low-density lipoprotein cholesterol
MACE	Major Adverse Cardiovascular Events
RPR	Residual platelet reactivity
